# A Rare Case of Recurrent Cystitis in a Primary Care Setting

**DOI:** 10.7759/cureus.48914

**Published:** 2023-11-16

**Authors:** Mariana M Silva, Ana B Costa, Carlos E Baptista

**Affiliations:** 1 Unidade de Saúde Familiar Cynthia, Regional Health Administration of Lisbon and Tagus Valley, Lisbon, PRT

**Keywords:** colovesical fistula, diverticular disease, rare etiology, escherichia coli, recurrent cystitis

## Abstract

Uncomplicated cystitis is common in women and typically presents with symptoms such as increased urinary frequency, dysuria, suprapubic pain, and urgency. *Escherichia coli* is the most frequently identified pathogen in these cases. Colovesical fistulas constitute an uncommon etiology of recurrent urinary tract infections, and they are even rarer in women due to the protective barrier provided by the uterus. Faecaluria and pneumaturia are the pathognomonic symptoms of these types of fistulas that help differentiate them from recurrent cystitis. While the gold standard imaging is the abdominopelvic CT scan, in some instances, MRI may be necessary to identify fistulous tracts. This case report describes a scenario of recurrent urinary tract infection caused by a colovesical fistula, in a woman with a history of diverticular disease. In contrast to uncomplicated recurrent cystitis, the treatment of the fistula is surgical.

The aim of this article is to raise awareness of this potential and rare cause of recurrent urinary tract infection encountered in a primary healthcare setting, in order to prevent the prescription of multiple cycles of ineffective antibiotic therapy in these patients and the consequent development of antimicrobial resistance, a global public health issue. Our intention is to alert general practitioners about the diagnosis of a rare cause of recurrent cystitis, the treatment of which is surgical and warrants referral to secondary care.

## Introduction

Acute uncomplicated cystitis typically presents with increased urinary frequency, dysuria, suprapubic pain, and urgency [1]. It occurs in 50-80% of women, accounting for a significant portion of antibiotic prescriptions in primary healthcare. Furthermore, 30-44% of women who have an episode of acute cystitis will experience a recurrence [2]. *Escherichia coli* is responsible for 66.4% of uncomplicated cystitis cases, and it can persist in the fecal flora after urinary elimination, leading to recurrent cystitis for up to three years after initial infection [3]. Recurrent cystitis is defined as three episodes of acute cystitis in the last 12 months or two episodes in the last six months [4].

In this report, we discuss a scenario of recurrent urinary tract infection, caused by a particularly uncommon etiology, whose treatment is surgical in most cases. The aim of this article is to raise awareness of this potential and rare cause of recurrent urinary tract infection presented in a primary healthcare setting, with a view to preventing the prescription of multiple cycles of ineffective antibiotic therapy and the consequent development of antimicrobial resistance, a global public health issue. We aim to alert general practitioners about the diagnosis of a rare cause of recurrent cystitis, the treatment of which involves a surgical approach and referral to secondary care.

This case report was previously presented at the V Jornadas Temáticas Patient Care - Urologia para Medicina Geral e Familiar, on September 29, 2022.

## Case presentation

An 82-year-old female with a medical history of hypertension and sigmoid colon diverticulosis, chronically medicated with 10 mg of lercanidipine, presented to the primary healthcare services on August 28, 2020, due to symptoms of suprapubic pain, dysuria, and hematuria. She was apyretic and had a negative renal Murphy's sign. The urinary Combur-Test® showed hematuria and leukocyturia with negative nitrites. She was empirically treated with a single dose of 3 g of fosfomycin and 200 mg of flavoxate every eight hours. A subsequent urine culture revealed a multisensitive *Escherichia coli*.

On^ ^September 10, the patient presented to the emergency department with a similar clinical picture, along with complaints of dark-colored urine and foul odor. Physical examination revealed tenderness in the hypogastric region. Laboratory results showed normal renal function, and negative inflammatory markers, and urine routine microscopic test showed increased hemoglobin levels, and increased leukocytes along with positive nitrites. A urine culture was not repeated, and she was discharged with a prescription of 500 mg of cefuroxime every 12 hours for seven days.

After 13 days, she was reevaluated in primary care due to persistent suprapubic discomfort, and dysuria, along with symptoms of pneumaturia and fecaluria, and reported the presence of grape seeds in her urine. A urine culture was ordered, which revealed *Escherichia coli* resistant to cefuroxime but sensitive to fosfomycin. A CT scan of the abdomen showed "the presence of gas within the bladder, thickening of the upper and left lateral bladder wall, in close contact with the adjacent colonic segment, without a clear cleavage plane" (images not available). She was urgently referred for a urology consultation.

**Figure 1:**
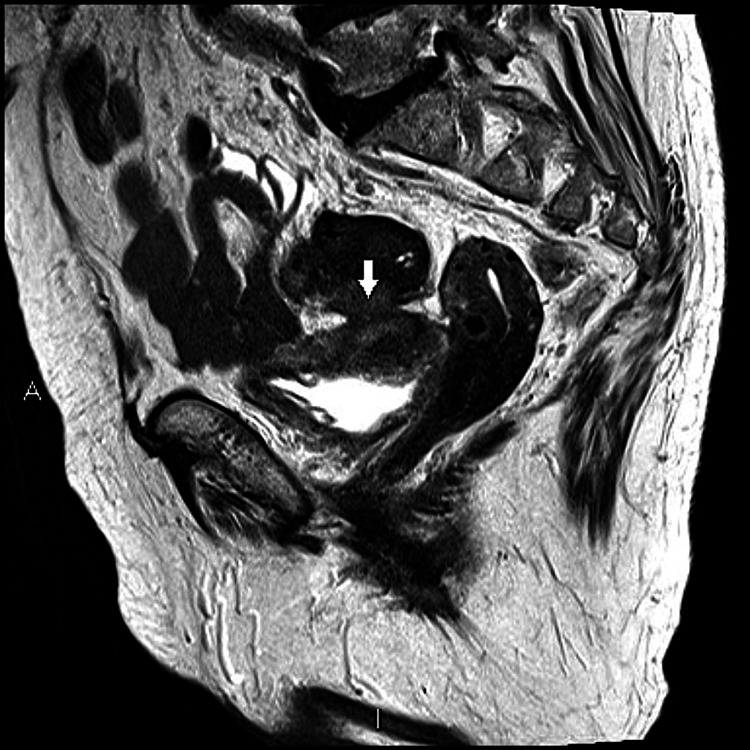
Colovesical fistula: sagittal section image The arrow points to the fistula

**Figure 2:**
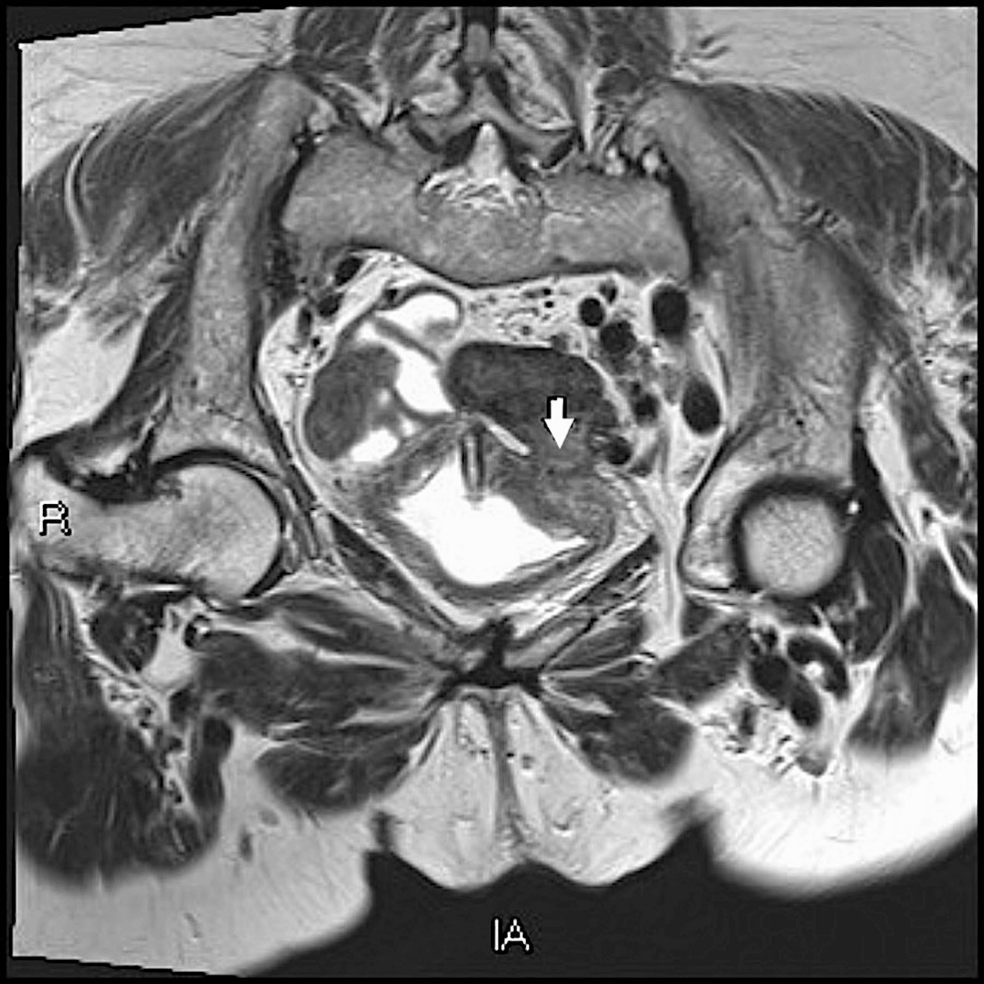
Colovesical fistula: coronal section image The arrow points to the fistula

On October 12, 2020, while still awaiting the urology appointment, she returned to the emergency department with symptoms of suprapubic pain, fecaluria, and unquantified fever for two days. Laboratory tests showed elevated inflammatory markers. She was admitted, and an MRI was performed, documenting a "colovesical fistula between the upper bladder wall and the lower sigmoid wall, probably of diverticular origin” (Figures 1, 2).

Colonoscopy revealed "multiple sigmoid diverticula", and cystoscopy showed no abnormalities. Finally, the patient was diagnosed with colovesical fistula as a complication of diverticulitis, and, on November 9, she underwent fistuloplasty with sigmoidectomy. She has experienced no new episodes of urinary tract infection so far.

## Discussion

Colovesical fistulas account for one out of every 3000 surgical hospital admissions. They are most commonly a complication of diverticulitis (65-79%), cancer (10-20%), or Crohn's disease (5-7%). Between 2 and 18% of patients with diverticular disease develop a colovesical fistula [5] and, in the case of diverticulitis, the incidence rises to 17-27%, making it an important and rare differential diagnosis in this patient with a history of diverticular disease and recurrent cystitis [6]. A colovesical fistula is defined as an abnormal connection between the colon and urinary bladder, as we observed in this patient (Figures 1, 2). It represents the most prevalent type of fistula (65%), followed by colovaginal fistulas (25%), coloenteric (7%), colouterine (3%), and colocutaneous fistulas. Men are more affected by colovesical fistulas than women, with a ratio of 3:1 to 2:1, which makes this patient's case even rarer. It is possibly attributed to the protective factors of uterine interposition and the broad ligaments between the sigmoid colon and the bladder. Also, they most frequently affect patients in their sixth or seventh decades of life [5].

Patients typically present with lower urinary tract symptoms, as in the presented case. Approximately 87.5% exhibit pathognomonic symptoms such as faecaluria or pneumaturia, and 30-90% report suprapubic pain [7]. The gold standard imaging is an abdominopelvic CT scan with oral or rectal contrast, reaching a sensitivity of 90-100% [5]. It easily detects intravesical air and contrast, although visualization of the colovesical fistula tract is achieved in only approximately 64% of cases [7]. MRI can accurately detect fistulous tracts without the need for direct contrast agents like in CT scans, achieving a sensitivity and specificity close to 100%. However, its limited accessibility in emergencies and high costs restrict its widespread diagnostic utility. In cases of fistulas resulting from diverticulitis, they are frequently found between the inferior wall of the sigmoid colon and the thickened, inflamed superior bladder wall, as in this case [8].

Patients in whom a colovesical fistula is confirmed on CT scan should subsequently undergo colonoscopy to ascertain the underlying etiology of the fistula and cystoscopy to rule out malignancy. Bladder cancer accounts for a small percentage (2-5%) of these cases. The primary approach to treatment of the fistula is surgical intervention, as observed in our case [5].

## Conclusions

In both primary and hospital healthcare settings, it is essential to reevaluate each case of recurrent cystitis before initiating a new antibiotic cycle and to ascertain the presence of any other etiological factor, including those requiring surgical intervention. This approach helps mitigate the overprescription of antibiotics and the resultant escalation of antibiotic resistance, a global issue. Furthermore, This case report alerts primary care physicians and those who work in the emergency department about a rare etiology of urinary tract infections.
